# One year prospective survey of *Candida* bloodstream infections in Scotland

**DOI:** 10.1099/jmm.0.47239-0

**Published:** 2007-08

**Authors:** Frank C. Odds, Mary F. Hanson, Amanda D. Davidson, Mette D. Jacobsen, Pauline Wright, Julie A. Whyte, Neil A. R. Gow, Brian L. Jones

**Affiliations:** 1 Aberdeen Fungal Group, School of Medical Sciences, Institute of Medical Sciences, University of Aberdeen, Aberdeen AB25 2ZD, UK; 2 Department of Medical Microbiology, Western General Hospital, Edinburgh EH4 10XU, UK; 3 Department of Medical Microbiology, Royal Infirmary, Glasgow G4 OSF, UK

## Abstract

A 12 month survey of candidaemia in Scotland, UK, in which every Scottish hospital laboratory submitted all blood isolates of yeasts for identification, strain typing and susceptibility testing, provided 300 isolates from 242 patients, generating incidence data of 4.8 cases per 100 000 population per year and 5.9 cases per 100 000 acute occupied bed days; 27.9 % of cases occurred in intensive care units. More than half the patients with candidaemia had an underlying disease involving the abdomen, 78 % had an indwelling intravenous catheter, 62 % had suffered a bacterial infection within the 2 weeks prior to candidaemia and 37 % had undergone a laparotomy. *Candida albicans* was the infecting species in 50 % of cases, followed by *Candida glabrata* (21 %) and *Candida parapsilosis* (12 %). Seven cases of candidaemia were caused by *Candida dubliniensis*, which was more prevalent even than *Candida lusitaniae* and *Candida tropicalis* (six cases each). Among *C. glabrata* isolates, 55 % showed reduced susceptibility to fluconazole, but azole resistance among other species was extremely low. Multilocus sequence typing showed isolates with high similarity came from different hospitals across the country, and many different types came from the hospitals that submitted the most isolates, indicating no tendency towards hospital-specific endemic strains. Multiple isolates of *C. albicans* and *C. glabrata* from individual patients were of the same strain type with single exceptions for each species. The high prevalence of candidaemia in Scotland, relative to other population-based European studies, and the high level of reduced fluconazole susceptibility of Scottish *C. glabrata* isolates warrant continued future surveillance of invasive *Candida* infections.

## INTRODUCTION

Bloodstream infections with *Candida* species arise in many different types of patient. Reports in recent years have varied between claiming an increase, a decrease and no change in incidence of candidaemia, but a recent review found the majority of surveys showed a stable incidence trend (Morgan, 2005) and very large North American studies point to a decrease in incidence of, and mortality due to, candidaemia through the 1990s (McNeil *et al.*, 2001; Trick *et al.*, 2002).

Few of the many surveys of candidaemia are population-based for a whole country or a geographical region. Surveys in different regions of the USA have shown dramatic differences in incidence, with an estimated 6 cases per 10^5^ population in Iowa (Diekema *et al.*, 2002), 7 cases per 10^5^ population in Connecticut (Hajjeh *et al.*, 2004), 8 cases per 10^5^ population in Atlanta and San Francisco (Kao *et al.*, 1999), and 24 cases per 10^5^ population in Baltimore (Hajjeh *et al.*, 2004). Outside the USA, population-based estimates of candidaemia incidence have mainly been much lower, ranging between 1.1 and 4.9 cases per 10^5^ population in surveys from Canada (Laupland *et al.*, 2005), Finland (Poikonen *et al.*, 2003), Iceland (Asmundsdottir *et al.*, 2002) and Norway (Sandven *et al.*, 2006). The striking exception is Denmark, with an incidence of 11 cases per 10^5^ population (Arendrup *et al.*, 2005). In the UK, a recent study based in 6 sentinel hospitals reported a rate of 3.34 cases of candidaemia per 100 000 bed days (Kibbler *et al.*, 2003).

Reasons for these incidence variations are unclear. One very recent study of Italian intensive care units (ICUs) shows a near-perfect association between annual incidence of candidaemia and defined daily doses of fluconazole (Bassetti *et al.*, 2006); however, the annual numbers are small, and the rise in incidence comes mainly from cases due to *Candida albicans* and *Candida parapsilosis*, rather than from species that typically show reduced fluconazole susceptibility (*Candida glabrata*, *Candida krusei*). Fluconazole prophylaxis has often been cited as a trigger for the emergence of low-susceptibility *Candida* species; the earliest example of such data was published more than 10 years ago (Price *et al.*, 1994). However, only *C. glabrata* fungaemia showed a statistically proven rise in incidence (as opposed to prevalence) in a survey that covered more than 3 million ICU patients over a 10 year period (Trick *et al.*, 2002) and an antifungal surveillance programme involving more than 13 000 bloodstream isolates of *Candida* species from 1992 to 2004 continues to show *C. krusei* as the most fluconazole-resistant *Candida* species, yet it accounts for only 2.3 % of all isolates (Pfaller *et al.*, 2006).

Changes in the medical and surgical management of patients over the past 15 years are likely to influence their vulnerability to haematogenous dissemination of *Candida* species that form part of their commensal flora. The mechanisms whereby procedures and agents used to manage patients with haematological malignancies predispose to dissemination of gastrointestinal fungal commensals have been described in fine detail (Blijlevens *et al.*, 2002). However, other gastrointestinal insults that arise as a consequence of abdominal surgery and ICU management procedures may have led to the now well-known shift of candidaemia away from its former association with neutropenia and into many other patient groups (Hajjeh *et al.*, 2004).

No published surveys of candidaemia have ever involved patients in Scotland, so we undertook a pilot, 1 year study of all cases of candidaemia identified by a positive blood culture for a *Candida* species. The antifungal susceptibilities of isolates were determined, and the epidemiological relationships of isolates of *C. albicans, C. glabrata* and *Candida tropicalis* were assessed by multilocus sequence typing (MLST).

## METHODS

Microbiology laboratories in all Scottish hospitals undertook to send to the Aberdeen Fungal Group laboratory subcultures of all yeasts isolated from blood cultures during the period March 1st 2005 to February 28th 2006. Isolates from catheter tips were not included. A short pro forma was provided to gather information on the patient with a yeast-positive blood culture, when this was available. The information requested included the patient’s age, sex, main underlying condition and neutrophil count at the time of the positive blood culture, and the presence or absence of an indwelling catheter, whether or not the patient was receiving total parenteral nutrition or corticosteroid therapy, other infections documented, and any antibacterial or antifungal therapy given in the 2 weeks before blood culture, the therapy given for the present episode of fungaemia and whether or not the intravenous (IV) catheter was removed as part of antifungal treatment. While full information was not available for all patients, the co-operation and enthusiasm of the participating physicians and laboratories was very high, and clinical data were obtained for the majority of cases. Most acute hospitals in Scotland participated and we are confident that very few instances of candidaemia were missed during the study period.

The isolates received were streaked to single colonies on CHROMagar Candida plates to facilitate detection of mixed cultures, and provide presumptive identification of *C. albicans/Candida dubliniensis, C. krusei* and *C. tropicalis* (Odds & Bernaerts, 1994). *C. albicans* was differentiated from *C. dubliniensis* by PCR determining the position of the *ITS1* intron (McCullough *et al.*, 1999). Other species were identified by standard morphological and physiological criteria. *C. parapsilosis* isolates were confirmed as *C. parapsilosis, Candida metapsilosis* or *Candida orthopsilosis* by PCR and restriction digestion (Tavanti *et al.*, 2005a). Isolates of *C. albicans, C. glabrata* and *C. tropicalis* were further characterized by MLST (Bougnoux *et al.*, 2003; Dodgson *et al.*, 2003; Tavanti *et al.*, 2005c). Isolates were characterized by their diploid sequence type (DST) for *C. albicans* and *C. tropicalis*, and by their (haploid) sequence type (ST) for *C. glabrata*. *C. albicans* isolates were assigned to MLST clades by reference to a UPGMA dendrogram drawn for the current database of 1391 *C. albicans* isolates (Odds *et al.*, 2007). Relatedness of *C. albicans* isolates that differed from each other in only one of the seven MLST gene loci was determined by eBURST analysis (Feil *et al.*, 2004) and isolates found to be related by this analysis were referred to as clonal clusters.

Antifungal susceptibilities of the isolates were measured by microdilution plate testing as previously described (Odds *et al.*, 1995). This method, including spectrophotometric determination of MIC end points from dose-response curves, is essentially the same as the European Committee on Antimicrobial Susceptibility Testing (EUCAST) antifungal methodology (Cuenca-Estrella *et al.*, 2002b, 2003, 2005) and all MICs were read at 24 h in conformity with EUCAST norms. Our method (Odds *et al.*, 1995), and the EUCAST method (Cuenca-Estrella *et al.*, 2002b), have been shown to give MICs for triazole antifungal agents that correspond well with those determined by the Clinical and Laboratory Standards Institute method (NCCLS, 2002).

## RESULTS

### Candidaemia: incidence and demographics

Over the course of 1 year, 300 yeasts were isolated in blood cultures from 242 patients in 19 Scottish hospitals. Two or more positive cultures on different days were obtained from twenty-three patients. In 19 of these instances, the multiple isolates were obtained within a maximum of 12 days apart (median 4 days, mode 1 day) and were regarded as single episodes of candidaemia. For the remaining 4 patients, second positive blood cultures were obtained 21, 24, 37 and 44 days apart. While these may therefore be regarded as separate episodes or cases of candidaemia, in all four instances the second isolate was either the same species (two patients) or the same MLST strain type (two patients) as the first isolate, suggesting relapse or recurrence of a single instance of candidaemia, rather than a newly incident case. Data from the most recent (2001) census (http://www.scrol.gov.uk/scrol/common/home.jsp) list the population of Scotland at 5 062 011. The population-based incidence of candidaemia in Scotland can therefore be calculated as 4.8 per 100 000 population per year. Data from the Information and Statistics Division, Common Services Agency, National Health Service Scotland, indicate 4 100 095 acute occupied bed days (AOBDs) for the period of the study. This results in a figure of 5.9 cases of candidaemia per 100 000 AOBDs.

Demographic and clinical information on the patients with candidaemia is summarized in Table 1. No information was provided for two patients; for the remainder, the information forms listed at least one item of data concerning the candidaemic patient. There were approximately equal numbers of male and female patients with candidaemia (Table 1); the 52 : 48 ratio of females to males matches the sex distribution of the population in the most recent Scottish census (http://www.scrol.gov.uk/scrol/common/home.jsp). Most cases of candidaemia arose in patients above the age of 50 years; there were 13 cases in children below the age of 5 (Table 1), of whom 4 were neonates (the median age of the paediatric patients was 2 years). Severe neutropenia [polymorphonuclear neutrophil (PMN) count <0.5×10^9^ cells ml^−1^] was very uncommon among the patients with candidaemia where the information was available; 95 % of the patients had neutrophil counts within normal limits (Table 1).

The presence of an indwelling IV catheter and recent treatment with antibacterial agents (most of which were broad-spectrum agents or combinations of narrow-spectrum agents) were factors common to three-quarters of the patients (Table 1). Almost 30 % of the patients were receiving total parenteral nutrition at the time of candidaemia, but only 12 % were undergoing treatment with corticosteroids (Table 1). Sixty-three cases of candidaemia (27.9 %) were detected in patients receiving intensive care. During the study period there were 49 805 occupied bed days (OBDs) on ICUs, equating to a rate of 1.26 cases of candidaemia per 1000 ICU OBDs.

### Underlying diseases in patients with candidaemia

Tables of underlying and predisposing factors in patients with candidaemia commonly represent patients with multiple factors more than once. To provide a clear idea of the types of conditions associated with candidaemia, Table 2 presents information on underlying diseases (excluding children less than 5 years old) by grouping patients according to the body system primarily affected by the underlying disease, where this could be determined. Diseases affecting abdominal systems, particularly the gastrointestinal tract, dominated the list of underlying diseases (Table 2). Recent abdominal surgery had been documented for 37 % of the patients overall; the 10 cases listed under this heading in Table 2 were those for which no other information was available. Moreover, 12.6 % of patients had solid tumours or sarcomas, making solid tumours a more common underlying condition than haematological malignancy in this set of patients.

The specific diagnoses listed on the data forms for ‘main underlying disease’ in adults with candidaemia mentioned a very wide range of associated conditions, extending to alcoholism (8 cases), IV drug abuse (5 cases), road traffic accidents (3 cases) and cystic fibrosis (2 cases), indicating that candidaemia can arise in a great number of clinical situations.

### Treatment of candidaemia episodes

Information on antifungal therapy was provided for 195 patients. Of these, 10 died before therapy could be commenced. A further 19 received no specific antifungal therapy. Among the 165 patients who were treated with antifungals, fluconazole was used in 127 patients (65.1 %) overall and in 125 adults, usually at doses of 400 or 800 mg per day (this detail was added voluntarily on 40 forms). Liposomal amphotericin B (AmBisome) was used in 29 patients (14.9 %) overall (24 were adults). Six patients were treated with voriconazole, three with amphotericin B deoxycholate formulation and one with caspofungin. Among the 125 adult patients who received fluconazole, 72 were infected with *C. albicans*, 23 with *C. glabrata* or *C. krusei* and 30 with other species. Among the 24 who received liposomal amphotericin B, the corresponding breakdown was 13 infected with *C. albicans*, 7 with *C. glabrata* or *C. krusei* and 4 with another species. The distributions of species infecting patients were therefore the same in the patients who received either agent (*χ*^2^ test, *P*=0.44).

Information on the removal of IV lines as part of the management of candidaemia was provided for 197 patients, of which line removal was inappropriate in 42 cases, either because the patient died before intervention or because the patient had not been catheterized. Among the 155 patients with candidaemia eligible for line removal, the IV catheter was removed in 120 (77.4 %) of cases.

### Mycology

*C. albicans* was the predominant species among the isolates from patients with yeast-positive blood cultures, recovered as the sole isolate in half of the cases (Table 3). *C. glabrata* was the second most common species isolated (21 %) followed by *C. parapsilosis* (12 %). None of the latter isolates proved to be *C. orthopsilosis* or *C. metapsilosis* by PCR/restriction digest. *C. dubliniensis* alone was isolated from 7 patients, and in combination with *C. glabrata* in a further patient. The prevalences of *C. krusei* (3 patients) and *C. tropicalis* (6 patients) were both very low.

MLST was performed on 152 *C. albicans* isolates (4 isolates from one patient had been identified at the originating hospital but were lost in shipment). Two or more isolates of the species were obtained from fourteen patients; they were from clades 1, 2, 3, 4 and 9. In 13 of these patients the isolates had either the identical DST by MLST or a very close relative of that DST, within the same clade (Odds *et al.*, 2007). In the 14th case, 5 of 6 consecutive blood isolates from a patient were all clade 4; the final isolate, obtained 7 days after the first isolate and 3 days after the 5th isolate, was clade 1, suggesting strain replacement in this patient. Table 4 shows the distribution of the *C. albicans* isolates between the various clades. Clade 16 is a new type, found by analysis of a much larger database (Odds *et al.*, 2007) than was previously published (Tavanti *et al.*, 2005b).

Previously, *C. albicans* clade 2 was the most common type found among isolates from the UK, followed by clade 4 (Tavanti *et al.*, 2005b; Odds *et al.*, 2007). In the present study clade 1 was the most commonly found, although clades 2 and 4 were also well represented (Table 4). Within each clade, particularly clades 1, 2, 4 and 6, a majority of isolates were so closely related they belonged to the same clonal cluster by eBURST analysis (Table 4). These clusters were, however, widely spread among different hospitals, suggesting the strain types were widespread across the whole Scottish population, rather than focussed within individual institutions. Four hospitals each contributed ten or more *C. albicans*-infected patients to the study. From 2 hospitals, which contributed 10 and 11 patients, the *C. albicans* isolates represented 5 clades, and from 2 others, which contributed 20 and 21 patients, there were 7 clades represented among the *C. albicans* isolates. We interpret these data as indicating no institutional preponderance of any particular subtype; nor was such a preponderance evidenced among hospitals that contributed fewer than 10 cases of *C. albicans* candidaemia.

Among the 50 patients who were the source of *C. glabrata* isolates, 27 STs were found, of which 15 had not been previously published (Dodgson *et al.*, 2003). Seven patients were the source of two or more *C. glabrata* isolates. In six instances, the identical ST was found in all isolates from the same patient; in one instance ST 10 was followed by ST 22 from a blood culture 4 days later. Phylogenetic clustering of large numbers of *C. glabrata* isolates has not yet been undertaken. The six isolates of *C. tropicalis*, from five hospitals, were all different DSTs.

### Antifungal susceptibility testing

The results of antifungal susceptibility testing (Table 5) showed a considerable prevalence of low azole susceptibility among isolates of *C. glabrata*. Only one and two *C. albicans* isolates fell into the susceptible dose-dependent (S-DD) MIC range when tested against fluconazole and itraconazole, respectively, and none were found in the resistant (R) category. By contrast, more than 60 % and 70 % of *C. glabrata* isolates showed reduced susceptibility (S-DD or R) to fluconazole and itraconazole, respectively. Even with voriconazole, MICs for 9 % of the *C. glabrata* isolates were above the ‘susceptible’ breakpoint. Low azole susceptibility was also prevalent among the ‘other species’ listed in Table 5, although the species with high azole MICs recorded included three isolates of *C. tropicalis*, where the MICs reflect a common artefactual trailing growth phenomenon (Arthington-Skaggs *et al.*, 2002; Rex *et al.*, 1998), and three isolates of *C. krusei*, which are inevitably resistant to fluconazole, and for which itraconazole and voriconazole MICs tend to be higher than for *C. albicans.* Five of the *Candida guilliermondii* isolates also showed reduced susceptibility to fluconazole and itraconazole.

## DISCUSSION

Our study has afforded a very thorough characterization of candidaemia in Scotland, even though it was set up only as a pilot survey without the management systems and full patient documentation that would normally be required for a multi-centre survey of invasive *Candida* infection. Our survey benefited from two factors. The first was the choice of a yeast-positive blood culture as the inclusion criterion. Because candidaemia represents an unequivocal diagnosis of invasive infection (Vazquez & Sobel, 2003) there can be no diagnostic ambiguity as may be encountered with other mycoses and other forms of *Candida* infection, particularly those involving growth of a *Candida* sp. from non-sterile sites. The second is the friendly community spirit of those involved in diagnostic microbiology in Scotland, which ensured an enthusiastic compliance with our request to send on yeasts isolated from blood. The 300 isolates we obtained from 242 patients in this survey, and our associated calculation of 4.8 cases per 100 000 population, therefore represents an accurate minimum estimate of the incidence of candidaemia in this part of the UK. The true incidence will be higher by an unknown factor since not all cases of invasive *Candida* infection will have been detected by blood culture, and, notwithstanding the high compliance of the participants, some positive blood cultures may not have been forwarded into our survey. Our incidence rate of 4.8 per 100 000 is at the high end of comparable studies from other European countries (Asmundsdottir *et al.*, 2002; Laupland *et al.*, 2005; Poikonen *et al.*, 2003; Sandven *et al.*, 2006) with the exception of Denmark (Arendrup *et al.*, 2005) and lower than has been recorded in surveys of four regions of the USA (Hajjeh *et al.*, 2004; Kao *et al.*, 1999).

The incidence rate of 5.9 per 100 000 AOBDs described here compares unfavourably with that of 3.34 described by Kibbler *et al.* (2003). Given the similar patient populations, it is difficult to explain the higher incidence in Scotland, although it is important to note that the data from England and Wales are now 7 years old. The proportion of candidaemia cases recorded on ICUs is markedly lower in our study (27.9 %) compared to the 45 % of Kibbler *et al.* (2003) and the 35 % of Marchetti *et al.* (2004). In contrast, our incidence rate of 1.26 cases per 1000 ICU OBDs is appreciably higher than the rate of 0.98 described by Blumberg *et al.* (2001). The differences in cases per occupied ICU bed day may be explained by differences in illness severity. Critical care encompasses both level 2 and 3 care, with level 3 patients requiring a greater degree of respiratory and other organ support. The difference noted between Scotland and England/Wales may be explained by the differences in relative distribution of level 2 and 3 beds within a critical care unit. While Blumberg’s data from the USA are more than a decade old and refer to surgical ICU patients only, differences in case mix may also explain the apparent discrepancy in the rate per patient day. The low proportion of cases in ICUs also serves to emphasize the importance of traditional risk factors such as presence of long lines, administration of broad-spectrum antibiotics and gastrointestinal surgery, that are increasingly present in a wide variety of patients outside the ICU setting.

Our data illustrate the difficulties of meaningful definitions of cases for candidaemia. In all but four instances where two or more blood culture isolates occurred, the positive repeat cultures were obtained within 12 days (and mainly within 7 days) of the first positive culture. However, in one patient with multiple positive blood cultures over 7 days, the final *C. albicans* isolate was unequivocally a different strain from the previous isolates, which means that case definitions of candidaemia may need to consider the species and strain types of isolates as well as the interval over which blood cultures were positive. In the four patients where a second blood culture was made from 3 to 6 weeks after the first, the species or strain type isolated was the same as the first.

The many factors predisposing to candidaemia are well known and include broad-spectrum antibiotic therapy, corticosteroid treatment, indwelling IV catheters, total parenteral nutrition, malignancy, neutropenia and recent abdominal surgery (Vazquez & Sobel, 2003). All are represented among the patients in the present study (Table 2). The predominance of abdominal pathologies underlying candidaemia in our patient group is emphasized by the data in Table 2, which attempts to focus on the single principle disease condition for which the patient was hospitalized. In 1969, Cohen *et al.* cultured samples aspirated through swallowed, radio-opaque tubes by healthy human volunteers to determine the carriage of *Candida* species in different parts of the gastrointestinal tract. They found the highest prevalence of positive aspirates in the lower portion of the tract, reaching 60 % in ileal aspirates and 70 % in the colon (Cohen *et al.*, 1969). In surgical patients at operation in a 1974 study, 47 % of patients carried a *Candida* sp. in the ileum and 65 % in the colon (Stone *et al.*, 1974). Similar surveys have not been conducted in more recent years, but these early data suggest that the typical frequencies of carriage found in oral and rectal or faecal samples underestimate the true prevalence of yeasts in the lower intestine. Diseases or surgical interventions that affect the intestine are therefore highly likely to create a conduit for yeasts to enter the bloodstream. Typical chemotherapeutic interventions in haematological practice also lower the barrier integrity of the gut wall, permitting yeasts to overgrow and enter the bloodstream (Blijlevens *et al.*, 2002).

The most recent recommendations for treatment of candidaemia include removal of IV catheters (this was done in 77 % of our catheterized patients) as well as systemic antifungal therapy (Pappas *et al.*, 2004). Fluconazole was the agent most widely used for treatment. However, our susceptibility testing data show that reduced susceptibility and resistance to fluconazole are common among isolates of *C. glabrata*, and it may prove simpler in clinical practice to use an alternative agent to treat *C. glabrata* candidaemia. *C. glabrata* isolates remain generally susceptible to amphotericin B, flucytosine and caspofungin (Table 5). When an isolate is found to be in the S-DD category, fluconazole dosing should be increased in an effort to achieve the AUC (area under the curve)/MIC ratio of 25 shown to be needed for eradication of infection in experimental pharmacodynamic studies (Andes, 2004; Andes & van Ogtrop, 1999). Our susceptibility data (Table 5) are similar to those of other studies published in recent years, and confirm low or negligible levels of antifungal resistance among *C. albicans* and *C. parapsilosis* isolates but a high level of reduced azole susceptibility among *C. glabrata* isolates (Diekema *et al.*, 2002; Pfaller *et al.*, 2002).

The prevalence of *C. dubliniensis* among our bloodstream isolates is worthy of attention. This species is often thought of as associated mainly with oral infections. However, it has been regularly reported as a cause of candidaemia since 1999 (Chan-Tack, 2005; Kibbler *et al.*, 2003; Meis *et al.*, 1999; Tortorano *et al.*, 2004) and one study even identified *C. dubliniensis* in over 16 % of cases of candidaemia (Fotedar & Al Hedaithy, 2003). Our own prevalence of *C. dubliniensis* (2.9 %) is slightly higher than that of a previous, smaller UK survey (Kibbler *et al.*, 2003), and it exceeded that of *C. krusei* and even *C. tropicalis* among candidaemia patients in Scotland. The prevalences of the three species found as major causes of candidaemia in Scotland are very similar to those reported in the 2003 Health Protection Agency (HPA) communicable disease surveillance survey of candidaemia in England, Wales and Northern Ireland (HPA, 2004). Recalculated to ignore yeasts submitted without identification, the HPA data show the prevalence of *C. albicans* as 60.0 % in England and Wales versus 50.0 % in our study, *C. glabrata* 18.1 % versus 20.7 % and *C. parapsilosis* 12.1 % versus 11.6 %. Between the surveys, it can be concluded that *C. albicans, C. glabrata* and *C. parapsilosis* currently account for more than 80 % of candidaemias in the UK, and that *C. tropicalis* has entirely lost its former position (Odds, 1988) as the second most common causative species.

*C. parapsilosis* is well recognized as a particularly common cause of candidaemia in neonates (Levy *et al.*, 1998; Rangel-Frausto *et al.*, 1999; Sandven, 2000). In our patient cohort there were only four neonates with candidaemia; three were infected with *C. albicans* and one with *Candida lusitaniae*. Among the other nine children aged 5 or less, three were infected with *C. parapsilosis*, and one with a mixture of *C. albicans*, *C. glabrata* and *C. parapsilosis*. The remainder were infected with *C. albicans*. The overall prevalence of *C. parapsilosis* among all species isolated from children under five was therefore 33.3 %. However, the number of paediatric cases was relatively so small that the association of *C. parapsilosis* with candidaemia in neonates and infants would not explain the overall high prevalence of the species even in the adult population. Recent surveys of candidaemia from Spain and Italy have shown prevalences of *C. parapsilosis* between 20 and 40 % (Almirante *et al.*, 2005; Cuenca-Estrella *et al.*, 2002a; Marco *et al.*, 2003; Pemán *et al.*, 2005; San Miguel *et al.*, 2005). This species is clearly emerging as a significant cause of candidaemia in Europe. A retrospective and unpublished PCR survey of 100 of our culture collection of isolates originally identified as *C. parapsilosis* revealed 6 were in fact *C. orthopsilosis* and 1 was *C. metapsilosis*. We were therefore surprised that none of the 28 isolates identified phenotypically in the present study was an example of either of the new species.

Strain typing of *C. albicans* and *C. glabrata* isolates by MLST confirmed previous evidence that individual patients usually carry a single strain type of a species in repeated isolates (Odds *et al.*, 2006). However, it is notable that single counter-examples were found, in two patients, each infected with one of the two species typed, showing that strain replacement is possible even in repeated blood cultures from patients with candidaemia. It is, perhaps, notable that 7 of the 12 patients with mixed species infections detected in blood cultures had undergone surgical procedures involving the bowel; as already stated, the significance of the bowel as a source for haematogenous dissemination of commensal *Candida* species cannot be underestimated.

Many of the *C. albicans* isolates collected from different hospitals across the whole extent of Scotland were found to be closely related by MLST, sometimes extremely so. This suggests that certain *C. albicans* strain types are more likely than others to be associated with human carriage and disease than others. The four main *C. albicans* strain clades described from all sources in previous studies (Blignaut *et al.*, 2002; Odds *et al.*, 2006; Pujol *et al.*, 2002; Soll & Pujol, 2003) remain the most commonly encountered in candidaemia in our study, and these and others were found across the range of hospitals submitting isolates. This indicates that the strain types found in candidaemia most probably reflect the same distribution as found in commensal isolates, and that hospitals do not serve as endemic foci of particular *C. albicans* strain types.

We did not attempt to collect data on mortality associated with candidaemia, since this study used a laboratory-based criterion for inclusion and the clinical information available was clearly collected with difficulty for some cases. A future, prospective study with dedicated staff able to access many more clinical data from patients with candidaemia and other types of invasive *Candida* infection would allow assessment of mortality and of other parameters relevant to the switch of *Candida* species from commensals to pathogens. This pilot study has shown that the incidence of candidaemia in Scotland is high, and the occurrence of *C. glabrata* as the second most common causative species, with a high proportion of isolates showing reduced azole susceptibility, indicating the potential value of continued future surveillance. Clinicians need to remain alert to the wide breadth of debilitating conditions in which candidaemia can arise.

## Figures and Tables

**Table 1. t1:** Selected demographic and clinical factors for patients with candidaemia

**Factor (no. of patients assessable)**	**No. of patients**
Sex (*n*=236)	
Male	114 (48.3 %)
Female	122 (51.7 %)
Age (years) (*n*=237)	
<5	13 (5.5 %)
5–29	8 (3.4 %)
30–49	44 (18.6 %)
50–69	87 (36.7 %)
70+	85 (35.9 %)
PMN count (*n*=192**)**	
<0.5×10^9^ cells l^−1^	7 (3.7 %)
0.5–1.0×10^9^ cells l^−1^	0 (0 %)
1.0–1.5×10^9^ cells l^−1^	3 (1.6 %)
>1.5×10^9^ cells l^−1^	182 (94.8 %)
IV catheter *in situ* (*n*=223)	175 (78.5 %)
Receiving total parenteral nutrition (*n*=201)	59 (29.4 %)
Receiving corticosteroids (*n*=190)	22 (11.6 %)
Infection within prior 2 weeks (*n*=220)	137 (62.3 %)
Antibacterial agents within prior 2 weeks (*n*=216)	160 (74.1 %)

**Table 2. t2:** Organ systems involved in the primary disease or condition affecting 214 adult patients who became positive for yeasts in blood cultures and for whom information on underlying conditions was available Systems are listed in decreasing order of prevalence; each patient is represented only once.

**System or disease type**	**No. of cases**
Gastrointestinal tract	48 (22.4 %)
Liver/spleen/pancreas/gall bladder	32 (15.0 %)
Multiple organ disease	22 (10.3 %)
Kidney/urinary tract	18 (8.4 %)
Cardiovascular	15 (7.0 %)
Lung	15 (7.0 %)
Haematological malignancy	13 (6.1 %)
Recent abdominal surgery (no other information given)*	10 (4.7 %)
Solid tumour (not involving another system listed)†	8 (3.7 %)
Sepsis	7 (3.3 %)
Bone/joint	5 (2.3 %)
Brain/meninges	4 (1.9 %)
Diabetes	4 (1.9 %)
Soft tissue infection	3 (1.4 %)
Other condition	10 (4.7 %)

*A total of 79/214 patients (36.9 %) had undergone recent abdominal surgery. †A total of 27/214 patients (12.6 %) had solid tumours.

**Table 3. t3:** Fungi isolated from blood cultures from 241 patients with fungaemia

**Species**	**Total no. of isolations**	**No. of patients**
*C. albicans*	156 (52.0 %)	121 (50.0 %)
*C. dubliniensis*	9 (3.0 %)	7 (2.9 %)
*C. glabrata*	68 (22.7 %)	50 (20.7 %)
*C. guilliermondii*	10 (3.3 %)	7 (2.9 %)
*C. krusei*	3 (1.0 %)	3 (1.2 %)
*C. lusitaniae*	6 (2.0 %)	6 (2.5 %)
*C. parapsilosis*	35 (11.7 %)	28 (11.6 %)
*C. tropicalis*	6 (2.0 %)	6 (2.5 %)
Other*	7 (2.3 %)	2 (0.8 %)
Mixed†		12 (5.0 %)

*Rhodotorula spp. (*n*=5), *Prototheca wickerhamii* (*n*=1), *Cryptococcus* sp. (*n*=1); two patients were each infected only with a *Rhodotorula* sp. †Two or more species isolated in a single blood culture (9 instances) or in consecutive blood cultures (3 instances): *C. albicans*+*C. glabrata* (6 cases), *C. albicans*+*P. wickerhamii* (1 case), *C. guilliermondii*+*Rhodotorula* sp. (1 case), *C. dubliniensis*+*C. glabrata* (1 case), *C. guilliermondii*+*C. parapsilosis* (1 case), *C. albicans*+*C. glabrata*+*C. parapsilosis* (1 case), *C. albicans*+*C. glabrata*+*Rhodotorula* sp. (1 case).

**Table 4. t4:** Summary of MLST data for blood isolates of *C. albicans*

**Clade**	**No. of patients (%)**	**Isolate in a single clonal cluster**	
		**No. of patients**	**No. of hospitals**
1	34 (26.6)	25	10
2	30 (23.4)	16	7
3	12 (9.4)	0*	0
4	16 (12.5)	12	7
5	2 (1.6)	0	0
6	6 (4.7)	4	4
8	4 (3.1)	0	0
9	5 (3.9)	2	2
11	9 (7.0)	2	2
12	1 (0.8)	–	–
16	7 (5.5)	3	2
Singletons	2 (1.6)	–	–

*There were three paired clonal clusters in clade 3; in each case the pair members came from different hospitals.

**Table 5. t5:** Susceptibility test results for bloodstream isolates of *Candida* species IC_50_ and IC_90_ are the concentrations required to inhibit 50 and 90 % of isolates, respectively. MIC breakpoints are according to Clinical and Laboratory Standards Institute method M27-A (NCCLS, 2002). No breakpoints have been set for amphotericin B or caspofungin.

**Species (no. tested)**	**Agent**	**MIC (μg ml^−1^)**			**No. S-DD or I (%)**	**No. R (%)**
		**Range**	**IC_50_**	**IC_90_**		
*C. albicans* (153)	Amphotericin B	0.063–1.0	0.50	1.0		
	Flucytosine	≤0.13– >64	≤0.13	0.25	2 (1.3)	1 (0.7)
	Fluconazole	≤0.13–16	0.25	0.50	1 (0.7)	0
	Itraconazole	≤0.032–1.0	≤0.032	≤0.032	1 (0.7)	1 (0.7)
	Voriconazole	≤0.032–1.0	≤0.032	≤0.032	0	0
	Caspofungin	≤0.032–1.0	0.063	0.13		
*C. glabrata* (66)	Amphotericin B	0.063–2.0	0.50	1.0		
	Flucytosine	≤0.13– >64	≤0.13	≤0.13	0	2 (3.0)
	Fluconazole	≤0.13– >64	16	32	36 (54.5)	4 (6.1)
	Itraconazole	≤0.032– >16	0.50	2.0	27 (40.9)	22 (33.3)
	Voriconazole	≤0.032– >8	0.50	1.0	3 (4.5)	3 (4.5)
	Caspofungin	≤0.032–1.0	0.25	0.50		
*C. parapsilosis* (35)	Amphotericin B	≤0.032–2.0	0.50	1.0		
	Flucytosine	≤0.13–32	0.13	0.25	0	2 (5.7)
	Fluconazole	≤0.13–2.0	0.5	1.0	0	0
	Itraconazole	≤0.032–0.25	≤0.032	0.13	2	0
	Voriconazole	≤0.032–0.063	≤0.032	0.063	0	0
	Caspofungin	≤0.032–8.0	1.0	2.0		
Other species* (35)	Amphotericin B	0.063–2.0	0.50	1.0		
	Flucytosine	≤0.13–32	≤0.13	2.0	0	1 (2.9)
	Fluconazole	≤0.13– >64	1.0	32	5 (14.3)	2 (5.7)
	Itraconazole	≤0.032–0.50	≤0.032	0.25	8 (22.9)	0
	Voriconazole	≤0.032–1.0	0.063	0.5	0	0
	Caspofungin	≤0.032–8.0	0.25	2.0		

I, Intermediate; R, resistant. *C. tropicalis (*n*=6), *C. guilliermondii* (*n*=10), *C. krusei* (*n*=3), *C. lusitaniae* (*n*=6), *C. dubliniensis* (*n*=9), *Cryptococcus* sp. (*n*=1).

## Abbreviations

- AOBD, acute occupied bed day
- DST, diploid sequence type
- ICU, intensive care unit
- IV, intravenous
- MLST, multilocus sequence typing
- OBD, occupied bed day
- PMN, polymorphonuclear neutrophil
- S-DD, susceptible dose-dependent
- ST, sequence type
